# Subterranean Retreat for Invalids

**Published:** 1840

**Authors:** 


### SUBTERRANEAN RETREAT FOR INVALIDS.

We hope not to be accused of a play upon words, in the title of this paragraph. Most of our readers have heard of the *Mammoth Cave*, near Green river in this State. Irregular and sinuous, it is said to extend for several miles, in ancient secondary limestone, to be of a uniform temperature throughout the year, to be free from all *excess* of humidity, and entirely exempt from any offensive or deleterious gases, insects, snakes or vermin. Such is the excavation, which our friend Dr. Croghan proposes to convert into a residence, during the heat of summer and the cold of winter, for such invalids, as may, at those seasons, regard a change of climate as necessary to their comfort. The enterprise is certainly novel, and we see no reason, why it should not in many instances, prove more salutary, than a visit either to the south or the north. Dr. C. being a man of science, will understand in what manner to make arrangements for the accommodation of valetudinarians, while, with the taste of a gentleman, he will provide suitably for the comfort and amusement of those, who in health, may visit this ancient resort of the extinct people of Kentucky, one of whom, completely mummyized, was found many years ago in a recess of the cavern.

